# Stem-Cell-Based Gene Therapy for HIV Infection 

**DOI:** 10.3390/v6010001

**Published:** 2013-12-24

**Authors:** Anjie Zhen, Scott Kitchen

**Affiliations:** Department of Medicine, Division of Hematology and Oncology, David Geffen School of Medicine at University of California, Los Angeles, CA 90095, USA; E-Mail: anjiezhen@gmail.com

**Keywords:** hematopoietic stem cell, HIV, gene therapy, CCR5, engineered immunity

## Abstract

Despite the enormous success of combined anti-retroviral therapy, HIV infection is still a lifelong disease and continues to spread rapidly worldwide. There is a pressing need to develop a treatment that will cure HIV infection. Recent progress in stem cell manipulation and advancements in humanized mouse models have allowed rapid developments of gene therapy for HIV treatment. In this review, we will discuss two aspects of HIV gene therapy using human hematopoietic stem cells. The first is to generate immune systems resistant to HIV infection while the second strategy involves enhancing anti-HIV immunity to eliminate HIV infected cells.

## 1. Introduction

The success of antiretroviral therapy in HIV infection has changed the landscape of HIV disease. Highly active antiretroviral therapy (HARRT), if initiated before advanced disease stages and if properly adhered to, can potently reduce the plasma HIV viral load to low or undetectable levels in most patients. This has changed what used to be a universally fatal disease to a potentially chronic disease. However, despite this success, antiretroviral therapy is not completely effective; chronic inflammation and immune dysfunction often persist and emerging evidence shows that there is cryptic viral replication in dispersed lymphoid organs during treatment [1]. These factors, along with toxic effects of antiretroviral drugs, have been shown to contribute to the increased risk of non-AIDS morbidity and mortality [2,3]. In addition, HAART regimen requires daily intake and many patients cannot maintain the high level of adherence necessary for viral control. Moreover, in resource-limited countries, it is difficult for many individuals to have continuous access to treatment. 

Given the limitations of the current therapeutic approaches and the absence of any effective vaccination strategy against HIV infection, there is a pressing need to develop a curative treatment. Hematopoietic stem cell (HSC) based gene therapies have emerged as a promising direction as these long-lived, self renewing progenitor cells could be modified to resist HIV infection [4,5]. If successfully engrafted, the modified HSCs would offer continuous, long-term production of genetically engineered cells that are resistant to HIV infection and/or have enhanced anti-viral activity to clear infected cells. If the host can be repopulated with a HIV-resistant hematopoietic system and eliminate all viral reservoirs, then a lifelong cure can be achieved. This review will focus on two aspects of HSC gene therapies for HIV infection: engineering HIV resistant cells and engineering cells that can target HIV infection. These two strategies represent the cutting edge of current gene therapy approached towards HIV infection and, alone or in combination with other strategies, have the potential to eradicate HIV. 

## 2. Engineering HIV Resistant Cells

**Figure 1:**
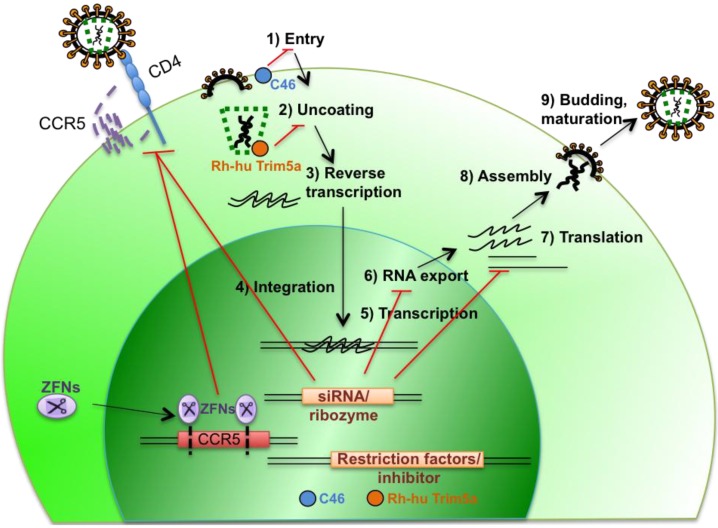
HIV lifecycle and strategies to engineer HIV-resistant cells.

By targeting different steps of HIV replication, several approaches are being developed to modify HSCs to render them resistant to HIV (Figure 1). These approaches can be grouped into three main strategies: The first targets cellular genes necessary for viral replication and for this review we will focus mainly on the recent successes in targeting CCR5 co-receptor necessary for viral entry; the second strategy directly targets HIV gene expression itself; and lastly, one that introduces genes that interfere with HIV replication, such as host restriction factors and fusion inhibitors. 

### 2.1. Targeting Expression of Cellular Genes that Are Essential for Viral Replication

CCR5 is a critical co-receptor for entry of HIV and CCR5-tropic viruses represent the majority of transmittable HIV-1 strains [6]. Individuals that are homozygous for a deletion of 32 base pairs of the CCR5 gene (Δ32) are largely resistant to HIV infection [7]. Individuals with a single copy of the Δ32 mutation also have slower disease progression once infected with HIV [8]. The identification of these individuals has made CCR5 an attractive anti-viral therapeutic target. For example, the small molecule antagonist Maraviroc, an allosteric inhibitor of CCR5/HIV interaction, was developed and proven to be a successful entry inhibitor [9]. However, antiviral resistance can develop to this drug; thus a more permanent strategy targeting CCR5 would be a more effective approach.

Targeting CCR5 as a potential treatment for HIV infection was further highlighted by the dramatic case of the “Berlin patient”, the first documented patient who was cured of HIV infection. This patient had acute myeloid leukemia and had received a bone marrow transplant from a donor who was homozygous for CCR5^Δ32/Δ32^. Following transplantation, the engrafted donor cells appeared to confer long-term control of HIV infection as the patient had no detectable HIV years after being off combination antiretroviral therapy [10,11]. However, this approach is highly impracticable as a common treatment strategy. The chance of finding a human leukocyte antigen-matched, CCR5^Δ32/Δ32^ homozygous donor is extremely rare (particularly among non-European groups) and allogeneic stem cell transplantation has significant associated morbidities and mortalities, limiting the application of this approach to patients with AIDS-associated malignancies as a last resort strategy. 

Gene therapies that modify autologous peripheral blood T cells or HSCs can be used to mimic the CCR5^Δ32/Δ32^ phenotype seen in the Berlin patient. In particular, genetically modified HSCs can be used to engineer a HIV-1 resistant immune system that would prevent ongoing viral infection. To that end, several gene therapy approaches are being tested to reduce CCR5 expression or disrupt the CCR5 gene. These include using RNA interference (RNAi) [12], using ribozymes [13] to reduce CCR5 RNA levels, or using intrabodies [14] and intrakines [15] to target the CCR5 protein directly. RNA interference can be achieved through the stable expression of CCR5 short hairpin RNA (shRNA) from lentiviruses. The recent study by Shimizu *et al*. demonstrated that the transduction of human CD34+ hematopoietic cells with a CCR5-specific shRNA expressing lentivirus appeared to have no major adverse effect on T cell development in humanized Bone Marrow/Liver/Thymus (BLT) mice. Down regulation of CCR5 was found in both human T cells and monocyte/macrophages in systemic lymphoid tissues and CCR5 tropic HIV-1 replication was effectively inhibited *ex vivo* [16]. 

An alternative strategy to block CCR5 expression is to directly disrupt its genomic sequence. Zinc finger proteins are sequence-specific DNA-binding proteins that can be coupled to a DNA endonuclease (termed Zinc finger nucleases, ZFNs) to cut DNA at specific sites. Importantly, ZFNs only need to be expressed transiently to achieve permanent disruption of target genes and are therefore less likely lead to immune elimination due to antigen presentation [17]. Initial studies by Perez *et al*. showed that transient expression of ZFNs can permanently and specifically disrupt 50% of CCR5 alleles in a pool of primary T cells [18]. Subsequent study using the humanized mouse model showed that CCR5 gene was disrupted in 17% of human CD34+ hematopoietic cells by ZFN via nucleofection and the modified cells were successfully engrafted in NOD/SCID/IL2rγ^null^ (NSG) mice. Cells with this modification successfully conferred resistance to R5-tropic HIV infection. Strikingly, mice transplanted with ZFN modified cells underwent rapid selection for CCR5^−/−^ cells during HIV infection and had significantly lower HIV-1 levels [19]. Based on these promising results, several clinical trials of ZFN targeting CCR5 are being evaluated in periphery T cells (clinicaltrials.gov: NCR00842634, NCT01044654). A recent study showed that ZFN nuclease delivered by a recombinant adenoviral vector effectively disrupted >25% *CCR5* gene in protein kinase C (PKC) activator pretreated HSCs isolated from granulocyte colony-stimulating factor (CSF)-mobilized adults and these cells underwent multi-lineage differentiation *in vitro* and *in vivo* [20]. 

A potential caveat of CCR5 gene therapy is that it may drive the selection of CXCR4 tropic viruses. A study targeting the CXCR4 molecule in peripheral CD4 T cells in the humanized mouse model using ZFNs showed that CXCR4 disrupted cells proliferated normally and provided protection from CXCR4 tropic HIV-1 [21]. However, CXCR4 is widely expressed on many cell types and plays important roles in multiple physiologic processes, such as T cell, B cell, cardiovascular and cerebellar development [22]. Thus, CXCR4 may not be a feasible target for HSC based gene therapy. 

### 2.2. Targeting HIV Gene Expression

HIV genes that are essential for viral replication can also serve as targets for HIV gene therapy. Among them, Tat and Rev are both transactivators and are critical for HIV-1 infectivity. In stem cells, HIV tat and its overlapping genes have been targeted using hammerhead ribozymes, which are small catalytic RNA molecules engineered to target specific RNA species. A Phase 2 gene therapy trial showed that patients that received a tat-vpr-specific ribozyme modified autologous CD34+ cells have higher CD4+ T cell counts compared to placebo group but have no statistically significant differences in viral load [23]. RNA decoys have also been used to inhibit Tat recognition of HIV TAR (trans-activating response region) and inhibit viral replication *in vitro* [2]. 

HIV Rev, the viral regulatory protein that allows the nucleus to cytoplasmic transport of unspliced viral RNA, can also be targeted in gene therapy. Dominant mutants or trans-dominant forms of Rev have been used to inhibit HIV replication in gene modified cells [2,3,24,25,26]. Decoy rev responsive elements (RREs) have also been tested as potential therapeutic targets and were shown to sequester Rev in a clinical trial [4,5,27]. 

Based on the pattern of HIV gene expression, different stages of the viral replication cycle can also be targeted by RNA interference (RNAi). All viral transcripts, including those encoding *tat*, *rev*, *gag*, *pol*, *nef*, *vif*, *env*, *vpr*, as well as the LTR, are susceptible to RNAi down regulation in cell lines [6,28]. A substantial problem of clinical application of RNAi is the virus’s high mutation rate and the ability to escape targeted therapy [7,29]. Therefore, as described previously, targeting cellular factors such as CCR5 by RNAi may be a more attractive gene therapy strategy than targeting viral transcripts. 

### 2.3. Introduction of Genes that Interfere with HIV Replication

An alternative strategy of HSC modification for HIV gene therapy is to introduce exogenous factors that will inhibit key steps of HIV infection. To inhibit HIV entry, a gp41-derived protein, C46, that is structurally similar to fusion inhibitor enfuvirtide, was developed and shown to effectively inhibit HIV entry [8,30]. Clinical trials in HIV infected patients on antiretroviral therapy with advanced disease and multidrug resistant viruses showed that C46 modified autologous T cells were well tolerated but had limited effects on plasma viral load [9,31]. 

Certain primate host restriction factors, such as Tripartite motif-containing protein 5 alpha (TRIM5α), apolipoprotein B mRNA editing enzyme, catalytic polypeptide-like (APOBEC) 3F and 3G, and tetherin are resistant to HIV-mediated inhibition and can prevent HIV infection; thus, they are potential candidates for gene therapy. For example, expression of rhesus tripartite TRIM5α, which binds to HIV capsid and interferes with the uncoating process, strongly protects human cells from HIV infection where the virus has evolved to escape restriction from human TRIM5α [10,32]. However, rhesus TRIM5α is not suitable for gene therapy due to its potential immunogenicity in humans. To overcome this potential antigenicity, a human-rhesus TRIM5α with a single amino acid substitution of the human TRIM5α sequence was created and has been shown to inhibit HIV infection [11,33]. CD34+ transduced with this chimeric TRIM5α allowed normal thymocyte differentiation in the SCID-hu thy/liv mouse and the modified human thymocytes were protected from HIV infection [12,34]. Another primate TRIM5α that can effectively block HIV infection is owl monkey TRIM5α that is fused with cyclophilin (cyp) A. A human TRIMcyp was developed and was shown to potently inhibit HIV in human T cells and macrophages *in vitro* and *in vivo* [13,35]. 

Despite the successful preliminary results, introduction of exogenous anti-HIV genes into HSCs raises several concerns. One is the expressed protein may be immunogenic and eventually removed by immune elimination. The other is insertion of genes with their own promoter raises risk of insertional activation of nearby genes [14,36]. Nonetheless, ongoing research of HIV restriction factors has revealed an increasing list of anti-HIV genes: from Vif-resistant APBOEC3G [15,37], to Vpu-resistant tetherin [16,38] that may be used as new strategies to inhibit HIV by gene therapy. 

### 2.4. Combination Therapy to Prevent Viral Escape

Due to the short life-cycle and high error rate nature of HIV replication, the virus mutates rapidly, resulting in high genetic variability. Antiviral drugs, when given as mono-therapy, usually only have a transient effect on HIV replication and current anti-retroviral therapies rely on the combined effects of antiretroviral drugs to defend against the development of resistance. Likewise, gene therapies that have a single HIV target are also potentially subject to the development of viral resistance. To this end, combined gene therapies that target multiple steps of viral replication are being developed. Several group have combined multiple anti-HIV genes into a single lentivirus vector [17,39,40,41,42]. One interesting approach recently reported by Digiusto *et al*. used a combination of a tat/rev shRNA, a TAR decoy and CCR5 ribozyme. Patients were treated for AIDS-related lymphoma and received lentiviral vector-modified autologous CD34+ hematopoetic stem cells through transplantation. The procedure was well tolerated and gene modified cells persisted for at least 24 months. However, the frequency of gene-modified cells in peripheral blood was too low (less than 0.2%) to see a clinical benefit for the patients. Due to obvious ethical concerns, the patients received concurrent HAART treatment and transplantation of both transduced and untranduced cells. Approaches that would allow selection of transduced cells before transplantation are under investigation and may give rise to a higher percentage of transduced cells for engraftment to achieve a sufficient therapeutic level for HIV-resistant cells [18,43,44]. 

## 3. Engineering anti-HIV Immunity

In addition to engineering cells to become resistant to new HIV infection, immunity also plays important roles in controlling HIV infection. Because HIV does not possess the immunogenicity to elicit protective responses and directly impairs key components of the immune system, most patients cannot develop effective immune responses to control HIV replication [19,45]. Viral persistence and latency in various reservoirs in the body are also key elements in maintaining the chronic nature of infection and various efforts are being made to attempt to purge or target these cells. Illustrative of the issue associated with an ineffective immune response coupled with an HIV purging strategy is seen in a recent study by Shan *et al*. [46]. They found that without proper immune stimulation, HIV-specific cytotoxic T lymphocytes cannot kill autologous latently infected resting CD4 T cells that were reactivated by histone deactylase inhibitor vorinostat *in vitro* [46,47]. This raises a critical issue that purging latent reservoirs may not be sufficient in eliminating HIV infection and the infected cell may return to latency because the host does not have the ability to generate an adequate response against HIV and eliminate HIV infected cells. 

Therapeutic vaccines were developed to boost and broaden HIV-1-specific CD4 and CD8 T cell responses, but with little success to date [22,48,49,50]. Conjugation of vaccines to anti-dendritic-cell antibodies showed promising result in inducing potent cytotoxic T lymphocyte responses [23,51] and is currently being investigated in a clinical trial (NCT01127464). Using autologous monocyte-derived dendritic cells (MD-DCs) pulsed with heat inactivated whole HIV as therapeutic vaccine also showed positive results in controlling plasma viral loads after antiretroviral therapy interruption [52]. Despite an initial reduction in viral loads, all patients that received the MD-DCs in this study had viral loads rebound back to detectable levels and overall antiviral immune responses to vaccine waned with time [52]. It is possible that preexisting immune responses in these HIV infected individuals may have already selected viral immune escape variants early in the infection. These studies highlight the difficulty in boosting existing host immunity for HIV elimination.

Another strategy to enhance host anti-viral immunity is to genetically modify peripheral blood cells with a molecularly cloned T cell receptor (TCR) or a chimeric molecule that can redirect cells to target HIV antigens. A peptide specific TCR from reactive T cells from infected individuals can be cloned and used to modify peripheral cells from the same patient [11,53,54,55]. In one study, a TCR from a patient that had a sustained and robust CTL response against HIV gag SL9 peptide was molecularly cloned. When introduced to primary CD8 cells via transfection, the genetically modified CD8 cells exhibited enhanced and polyfunctional immune responses against viral antigen and an increased ability to control HIV infection [55]. A Phase 1 clinical trial (NCT00991224) is being carried out to study the effect of redirecting autologous T cells with SL9 TCR for HIV gene therapy [56]. However, there are several pitfalls for redirecting peripheral immunity. First, *ex vivo* manipulation of the cells significantly impacted the lifespan and function of the cells and therefore the modified cells had limited effects once re-infused back to the body. Another caveat or risk in these studies was that modifying peripheral T cells with a cloned TCR may have resulted in mispairing with endogenously expressed TCR α and β chains. Cross pairing of TCRs may produce self-reactive T cells since gene modified cells are not subject to normal thymic selection for peripheral tolerance. Lastly, many identified highly effective HIV-specific cytotoxic T lymphoctyes are restricted by uncommon HLA class I alleles such as HLA-B*27 and HLA-B*57 and cannot be universally applied [22]. 

To circumvent the HLA restriction of these molecularly cloned TCRs, an HIV specific chimeric antigen receptor (CAR) was developed that contains the extracellular domain of CD4 receptor (which binds to HIV *env*) and intracellular ζ chain of the T cell receptor (which mediates T cell activation). CD4 CAR -modified T cells inhibit viral replication and kill HIV infected cells *in vitro* and were reported to have prolonged survival *in vivo* [57,58]. However, CD4 CAR-modified T cells are susceptible to HIV infection and the level of genetically modified cells in patients drops one year after blood transfusion, resulting in a limited anti-HIV effect [58]. 

Stem cell based anti-HIV immune therapy, in contrast, could potentially bypass many of the problems that previous studies and clinical trials have encountered and have several advantages. A stem cell based “redirection” approach with a molecularly cloned TCR or CAR would allow proper thymic selection of the modified cells and exclusion of endogenous TCR surface expression, eliminating the risk of generating self-reactive TCR through mispairing [59]. In addition, stem cell based gene therapy would allow long-lived and renewable immunity capable of continuously generating anti-viral cells that could overcome the barriers that prevent HIV eradication. Recently, we showed that humanized mice transplanted with human HSCs genetically modified with an anti-HIV SL9 TCR were able to significantly suppress viral replication compared to control mice following challenged with HIV [60]. The genetic modification of HSCs with a TCR allows the cells to differentiate *in vivo* and the TCR modified stem cells differentiated to mature CD8+ cells in multiple tissues [60]. This study highlighted the potential of redirecting anti-HIV immunity using hematopoietic stem cells. Current advances in identifying antigen specific TCR may make it possible to molecular clone multiple TCR from each patient rapidly [61]. The cloned TCR, which match the patient’s HLA type and viral genome, can then be optimized and used to genetically modify patient’s HSCs to allow development of enriched and sustained modified CD8+ T cells capable of targeting and potentially eradicating HIV infected cells. 

## 4. Conclusions

As the safety of gene delivery has improved significantly, gene therapy has become an increasingly appealing strategy for treating HIV disease [22]. In particular, research on HSC-based anti-HIV gene therapy has shown promising results in attempts to cure HIV disease. The use of genetically modified HSCs can potentially generate long lasting, renewable immune cells that are programmed to be either resistant to HIV infection or have the enhanced ability to eliminate HIV infected cells. Efforts are also directed at combining both of these approaches and attempting to protect engineered HIV-specific immune cells from HIV infection. It is expected that the safety of genetically modifying HSCs will continue to improve as more development occurs in this approach from other studies, such as cancer research [62]. With increasingly more advanced techniques in collecting, expanding, and genetically manipulating HSCs, HSCs based gene therapy may ultimately offer a curative therapy for HIV infection. 
